# Detection and natural history of HPV infection of oral cavity and tonsils – a systematic literature review

**DOI:** 10.1186/s12885-025-14547-5

**Published:** 2025-09-01

**Authors:** Margaret Maltseva, Charlotte Klasen, Nora Wuerdemann, Malte Hannich, Jens Peter Klussmann, Ulrike Wieland

**Affiliations:** 1 Internal Medicine C, Medical Faculty, University of Greifswald, Ferdinand-Sauerbruch-Straße, Greifswald, 17475 Germany; 2 Department of Otorhinolaryngology, Head and Neck Surgery, Medical Faculty, University of Cologne, Kerpener Strasse 62, Cologne, 50937 Germany; 3 Center for Molecular Medicine Cologne (CMMC), University of Cologne, Faculty of Medicine and University Hospital Cologne, Robert-Koch-Str. 21, Cologne, 50931 Germany; 4 Department I of Internal Medicine, Center for Integrated Oncology Aachen Bonn Cologne Duesseldorf, Faculty of Medicine and University Hospital Cologne, University of Cologne, Kerpener Strasse 62, Cologne, 50931 Germany; 5 Institute of Virology, National Reference Center for Papilloma - and Polyomaviruses, University of Cologne, Faculty of Medicine and University Hospital Cologne, Fuerst-Pueckler-Str. 56, Cologne, 50935 Germany

**Keywords:** Human Papilloma Virus (HPV), Oral HPV prevalence, Epidemiology, HPV vaccination

## Abstract

**Background:**

Human papillomavirus (HPV)—associated oropharyngeal cancer (OPC) is increasing, with HPV16 being the most prevalent type. Persistent oral HPV infections play a causal role in the pathogenesis of these cancers. The objective of this systematic review was to summarize current data on oral HPV prevalence in the general population and in people living with HIV (PLWH), possible effects of prophylactic vaccination and optimal sampling methods for the detection of HPV in the oral cavity.

**Methods:**

We searched Medline and Livivo for publications on oral HPV prevalence in cohorts > 1000 individuals (> 100 individuals for cohorts of PLWH) released between January 2012 and October 2024. In total, 51 original studies and meta-analyses were included in this review.

**Results:**

Overall prevalence of oral HPV infection in general population/healthy individuals was between 0.67 and 11.89% (mean 5%) and was higher in males than in females. Prevalence of oral high-risk HPV ranged between 0.5 – 4.7%. The most prevalent HPV-type detected was HPV16. Risk factors for oral HPV infection comprised older age, male sex, the number of lifetime (oral) sex partners, smoking, drug abuse, oral health and concurrent genital HPV infection. Compared to the general population, higher oral HPV prevalence rates were detected in PLWH (2 – 40%, mean 20%). HIV infection has been established as an independent risk factor for oral HPV infection irrespective of gender or sexual orientation. Concerning prophylactic HPV vaccination of adolescents and young adults there is evidence from clinical and epidemiological studies showing prevention of oral HPV infection in vaccinated individuals.

**Conclusions:**

Oral HPV-DNA can be found in 1–12% of the general population, more frequently in men than in women. PLWH have an increased oral HPV prevalence compared to the general population. Since prophylactic HPV vaccination is associated with a significant reduction in vaccine-type oral HPV prevalence, high vaccination rates in children and adolescents are important to counteract the rising incidence rates of HPV-associated OPC in the future. Comprehensive research on oral HPV clearance and persistence and on optimal sampling methods is still pending.

## Background

Human papillomaviruses (HPV) are small non-enveloped double-stranded DNA-viruses that can infect multilayered squamous epithelia of the skin and mucous membranes. Currently more than 230 HPV-types are classified. Of those, more than 40 HPV-types of the genus alpha-papillomavirus infect the anogenital tract and the oral cavity [1, 2]. Based on their oncogenic potential, alpha-HPV are divided into low-risk (LR) and high-risk (HR) HPV-types. The latter group comprises 12 cancerogenic HPV-types with HPV16 being the most oncogenic type. Further HPV-types are classified as probably or possibly carcinogenic [3, 4]. LR-HPV-types such as HPV6 and HPV11 can cause benign anogenital warts, low-grade anogenital dysplasias, oral papillomas and respiratory papillomatosis [5]. Persistent infections with HR-HPV-types can lead to anogenital dysplasia and cancer such as cervical, vaginal, vulvar, penile and anal cancer as well as to oropharyngeal carcinoma (OPC).

Both, the proportion of OPC attributable to HPV and the incidence of HPV-associated OPC are rising. Recent studies have shown that the proportion of OPC attributable to HPV has significantly increased in the last decades, from below 20% in the 1980 s to 40–80% in 2000–2017, with wide geographic variation [5–8]. Furthermore, the incidence of OPC has increased sharply in high-income countries, especially in males, but also in women [8–10]. In the United States the annual number of HPV-associated OPC in men has surpassed the annual number of cervical cancers in women in recent years (15,540 HPV-associated OPC in males and 3,460 in females compared to 12,015 cervical cancers in 2016) [10–12]. The mechanisms driving HPV-associated OPC development are not fully understood. In contrast to anogenital HPV-induced cancer, a distinct precursor lesion has not yet been described for OPC. Regarding the anatomical location of HPV-induced OPC, HPV-DNA was found in 56% (95% CI 54–58%) of tonsillar, 40% (95% CI 38–43%) of base of tongue, 19% (95% CI 16–22%) of posterior wall and 12% (95% CI 9–15%) of soft palate carcinomas [13]. This demonstrates that HPV-associated OPC are predominantly found in lymphoepithelial subsites such as the palatine tonsils and the base of tongue.

HPV16 is the most prevalent HPV-type found in OPC and can be detected in approximately 90% of HPV-associated OPC. In the remaining 10% other HR-HPV-types, for example HPV18, HPV35 or HPV45 can be found [14]. Regarding oral HPV prevalence in non-cancer patients there is still limited data on where most HPV-infections are located (oropharynx vs. oral cavity). The most frequently used method for collecting oral samples for HPV-DNA detection is oral rinse or gargle. By this collection method it cannot be determined where the detected HPV-DNA was located (oropharynx vs. oral cavity). Studies comparing different sampling methods are scarce. The primary goal of this review is to summarize current data on oral HPV prevalence and to present differences in oral HPV prevalence across various population groups by gender, age, HIV status, and geographic region. Secondary goals were to outline current knowledge of oral sites of HPV infection, risk factors for oral HPV infection, the effects of prophylactic HPV vaccination on oral HPV prevalence and optimal sampling methods for the detection of oral HPV.

## Methods

**Fig. 1:**
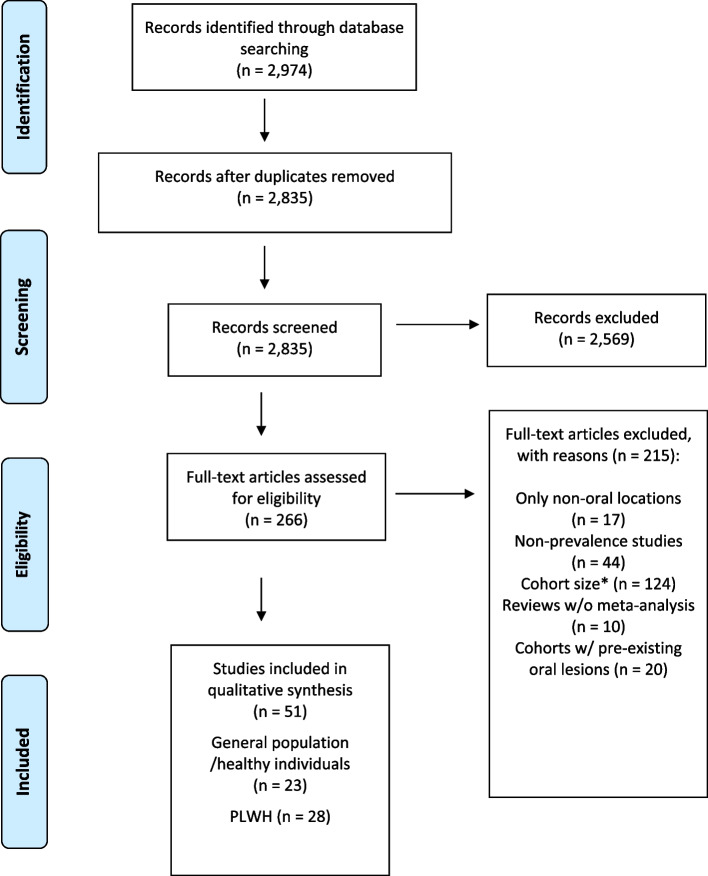
Preferred Reporting Items for Systematic Review and Meta-Analysis (PRISMA) flow diagram for studies concerning oral HPV prevalence in the general population and in PLWH. w/o, without; w/, with For each of the 215 excluded full-text articles only one (the most relevant) reason for exclusion is listed in the figure. *Concerning cohort size as a reason of exclusion, 16 full-text articles on the general population had a participant number < 100, 72 had participant number between 100 and 499, and 17 had a participant number between 500 and 999. 19 articles on PLWH had a participant number < 100

For this systematic review we performed a literature search following the criteria of “Preferred Reporting Items for Systematic Review and Meta-Analysis” (PRISMA) [15]. For Tables 1, 2 and 3, a systematic literature search was carried out to identify all relevant articles using the National Institutes of Health PubMed and Livivo search engines for English language. Only articles published between January 1, 2012 and October 1, 2024 were considered (Fig. 1).

For data on oral HPV prevalence in the general population the advanced search builders of the above-mentioned search engines were used. Research articles were selected by using the following terms: “HPV” or “papillomavirus”, “oral” or “oropharyngeal”, and “prevalence”. Only articles with available English language full text about human subjects were considered. 2974 records (2,798 search results from the PubMed database and 176 search results from the Livivo database) were screened by title and abstract and duplicates were removed (Fig. 1). All articles not covering oral or oropharyngeal alpha-HPV prevalence or only dealing with beta- or gamma-HPV were manually excluded. 266 articles were assessed for eligibility. For oral HPV prevalence in the general population or in healthy/non-immunosuppressed individuals only studies with ≥ 1000 individuals were included. This large sample size was chosen because of a lower risk of bias in larger population-based studies. In addition, we assumed that larger, better-planned studies are more likely to provide all the relevant data needed for the review. Different publications referring to the same large population-based study (e.g. NHANES from the United States) were only included if they addressed different research questions such as different risk factors or covered different data collection time frames. Due to the small number of large prevalence studies on PLWH, studies with ≥ 100 individuals were included for this patient group. For both, the general population/healthy individuals and PLWH, only studies reporting prevalence data in individuals without oral lesions were considered. Studies on subjects with benign or malignant oral lesions were excluded. Studies not complying with the previously established criteria, reviews without meta-analysis, case–control studies, case series and case reports were excluded (Fig. 1). Studies assessing concomitant HPV infection in other body sites were included as long as data on oral HPV prevalence was presented for the entire population studied and not only for the subjects with concomitant anogenital HPV infection. All searches were performed by three independent reviewers (M.M., C.K. and U.W.), discrepancies were solved through consensus. In total, 23 original studies and meta-analyses on oral HPV prevalence in the general population/healthy individuals and 28 original studies and meta-analyses in PLWH were included in this review (Fig. 1, Tables 1 and 2). No overlapping studies concerning both general population/healthy individuals and PLWH and fitting the search criteria were identified.

Quality assessment was performed using the STROBE Checklist of items that should be included in reports of cohort studies [67]. Quality assessment was completed by two reviewers (M.M. and C.K.). A study was assigned a low risk of bias if ≥ 20 items of the STROBE checklist were fulfilled. 15–19 was considered a medium risk of bias and ≤ 14 a high risk of bias.

Data extraction was performed by M.M. and C.K. Discrepancies were solved by consensus. Data extracted included general information (publication title, authors, year of publication, study type), study characteristics (years of recruitment, sample size), participant characteristics (age, country or region), the method of sample collection, percentage of HPV-positive and HPV-negative participants for any HPV and for high-risk HPV, most prevalent HPV type and limitations according to the authors of the study, if available. Risk factors for oral HPV-DNA prevalence were only considered if statistical significance had been determined in univariate analyses or in multivariate models. Contradicting results are explicitly discussed.

Studies comparing sampling methods (Table 4) and studies on solid organ transplant recipients (SOTR) and tonsillectomy patients were discussed separately irrespective of the cohort size due to the lack of larger cohorts. Data on persistent HPV infections, clearance, and incidence are not included in the tables, but are reviewed in the text.

RStudio (R version 2023.09.1) was used for statistical and graphical processing of the data.

## Results

### Oral HPV prevalence in the general population/healthy individuals

Almost 280 studies on oral HPV prevalence in the general population/healthy individuals have been published and a wide range of oral HPV prevalence rates have been reported. In Table 1 all meta-analyses and large population-based studies (> 1000 participants) are summarized (*n* = 23). All studies shown in Table 1 had a low risk of bias.

**Fig. 2:**
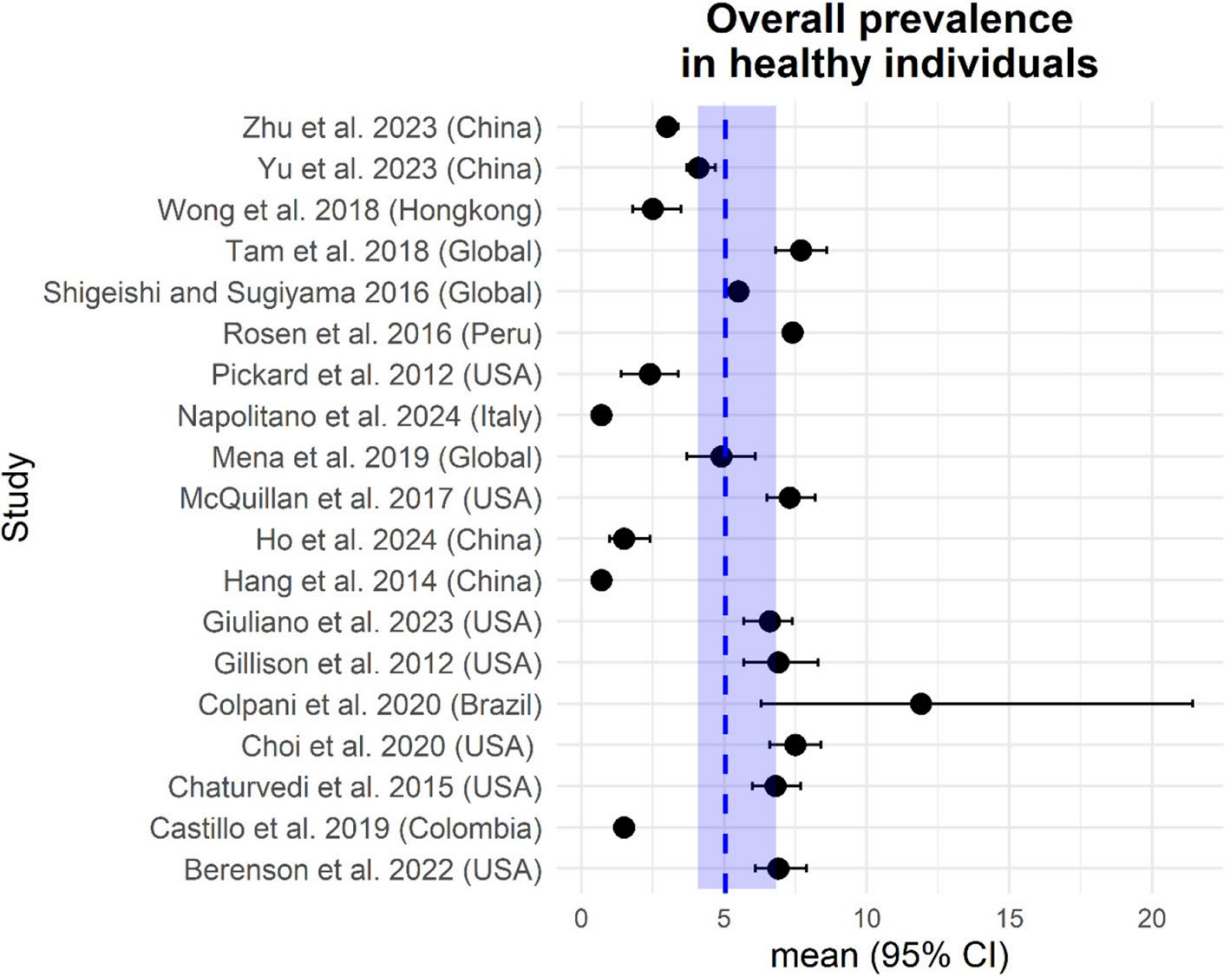
Forrest-plot summarizing the overall oral HPV prevalence from studies (2012–2024) with healthy individuals reporting data on males and females. The blue dotted line represents the mean prevalence over all studies. The shaded blue area marks the upper - and lower confidence interval

Overall HPV prevalence rates in males and females are between 0.67 and 11.89%. Figure 2 shows all studies from Table 1 that report data on overall HPV prevalence in the general population/healthy individuals comprising males and females. In contrast to all other large studies included, Hang et al. and Napolitano et al. estimated a prevalence rate below 1% in their cross-sectional studies [17, 35]. However, the authors themselves listed several limitations of their studies that could explain the low prevalence rates found such as the low participation of younger individuals or suboptimal sampling methods. With this exception, the differences in prevalence in the respective sub-cohorts of overall HPV, HR-HPV and HPV16 prevalence are small across all large-scale studies (Table 1 and Fig. 2).

**Table 1:** Oral HPV prevalence in healthy individuals as reported in large studies and meta-analyses. Overall HPV prevalence rates (males + females) are between 0.67 and 11.89%

Study	Type of study and population	Years of recruitment	No. of oral samples	Type of samples	Age range (years)	Region	Overall (alpha) HPV prevalence			HR-HPV prevalence°			Most prevalent HR-Type°	Limitations/risk of bias
							M + F; % (95% CI)	M; % (95% CI)	F; % (95% CI)	M + F; % (95% CI)	M; % (95% CI)	F; % (95% CI)		
Ho et al. 2024 [16]	Cross-sectional General population	2021–2023	2323	OR	R:18–75	China	1.5 (1.0–2.4)	1.5 (0.9–2.3)	1.5 (0.9–2.3)	0.7 (0.5–1.0)	0.7 (0.3–1.2)	0.8 (0.4–1.5)	52	1) 4)
Napolitano et al. 2024 [17]	Cross-sectional Youth Adults	2022–2023	1182	OR	R: 18–30	Italy	0.7 (NM)						NM	1)
Giuliano et al. 2023 [18]	Cross-sectional General population	2021–2022	3196	OR	R:18–60	USA	6.6 (5.7–7.4)	9.1 (7.6–10.6)	4.6 (3.6–5.5)	2.0 (1.5–2.5)	3.3 (2.4–4.2)	1.0 (0.3–1.5)	16	1)
Zhu et al. 2023 [19]	Cross-sectional General population (DLCC cohort)	2021	9867	SW	≥ 20 X̅:_:_46.6	China	3 (2.7–3.4)	3.6 (3.0–4.2)	2.7 (2.4–3.1)	1.3 (1.1–1.5)	1.7 (1.2–2.1)	1.2 (0.9–1.4)	16	4) 5)
Yu et al. 2023 [20]	Cross-sectional General population	2021	4226	SW	≥ 20	China	4.08 (3.7–4.7)	5.0	3.6	1.92 (1.5–2.3)			16	3)
Berenson et al. 2022 [21]	Cross-sectional General population (NHANES cohort)	2011–2016	9437	OR	R: 18–59	USA	Total 6.9 (6.1–7.9)	Total 10.9 (9.4–12.3)	Total 3.2 (2.6–4.0)	Total 4.2 (3.6–4.9)	Total 7.0 (5.9–8.2)	Total 1.6 (1.2–2.2)	NM	3) 4)
							Vacc. 4.2 (3.2–5.5)	Vacc. 9.5 (6.0–14.7)	Vacc. 2.7 (1.8–4.1)	Vacc. 2.7 (1.9–3.9)	Vacc. 7.6 (4.3–12.9)	Vacc. 1.3 (0.7–2.4)		
							Unvacc. 7.2 (6.3–8.3)	Unvacc. 10.9 (9.4–12.4)	Unvacc. 3.3 (2.6–4.3)	Unvacc. 4.4 (3.7–5.1)	Unvacc. 6.9 (5.8–8.3)	Unvacc. 1.7 (1.2–2.3)		
Colpani et al. 2020 [22]	Systematic review Meta-analysis General population	2006–2017	2,494	NM	R: 14–79	Brazil	11.89 (6.26–21.43)			4.69 (0.23–50.72)			NM	3) 6)
Choi et al. 2020 [23]	Cross-sectional General population (NHANES cohort)	2011–2014	8,229	OR	X̅: 44.0 R: 18–69	USA	7.5 (6.6–8.4)						NM	1) 3) 4)
Mena et al. 2019 [24]	Systematic review Meta-analysis General non-high-risk population	1995–2015	28,544	VAR	R: NM	Global	4.9 (3.7–6.1)	4.3 (2.7–6.4)	3.8 (2.6–5.2)	2.6 (1.7–4.7)			16	3) 6)
Schlecht et al. 2019 [25]^a^	Longitudinal Cohort Adolescent health clinic patients	2007–2017	1,259 (1,067 vacc.)	OR	X_med_: 18 R: 13–21	USA			6.2 (4.9–7.7)			1.7 (1.0–2.5)	Vacc. 58	2) 3)
													Unvacc. 16	
Bettampadi et al. 2019 [26]	Cohort Healthy men (HIM cohort)	2005–2009	3,098	OR	R: 18–73	Brazil		8.7 (7.1–10.4)			5.3 (4.1–6.7)		16	3)
						Mexico		10.0 (8.3–12.1)			7.3 (5.7–9.0)			
						USA		7.6 (5.9–9.5)			5.4 (4.0–7.0)			
Castillo et al. 2019 [27]	Cross-sectional High school students	NM	1,784 (944 vacc.)	OR	R: 14–17	Colombia	1.51 (NM)	2.28 (NM)	Vacc. 0.74 (NM)				16	3)
									Unvacc. 3.16 (NM)					
Tam et al. 2018 [28]	Systematic Review Meta-analysis General population	1995–2017	56,600	VAR	≥ 18	Global	7.7 (6.8–8.6)	9.3 (6.4–12.5)	5.5 (4.5–6.6)	3.5 (2.5–4.7)			NM	4) 6)
Wong et al. 2018 [29]	Cross-sectional General population	NM	1,426	OR	R: 18–64	Hong Kong	2.5 (1.8–3.5)	3.5 (NM)	1.6 (NM)	0.8 (0.4–1.4)			16	3) 5)
Sonawane et al. 2017 [30]	Population based (NHANES cohort)	2011–2014	9,134	OR	R: 18–69	USA		Total 11.5 (9.8–13.1)	Total 3.2 (2.7–3.8)		Total 7.3 (6.0–8.6)	Total 1.4 (1.0–1.8)	16	1) 3) 5)
								Vacc. 10.3 (4.8–15.8)	Vacc. 3.8 (2.1–5.5)		Vacc. 7.7 (2.0–13.4)	Vacc. 1.5 (0.3–2.6)		
								Unvacc. 11.5 (9.5–13.5)	Unvacc. 3.1 (2.2–4.0)		Unvacc. 7.3 (5.7–8.9)	Unvacc. 1.5 (1.0–2.0)		
McQuillan et al. 2017 [31]	Cross-sectional (NHANES Cohort)	2011–2014	NM ^b^	OR	R: 18–69	USA	7.3 (6.5–8.2)	11.5 (9.9–13.2)	3.3 (2.7–3.8)	4.0 (3.3–4.7)	6.8 (5.6–8.1)	1.2 (0.9–1.6)	NM	NM
Shigeishi and Sugiyama 2016 [32]	Systematic Review Meta-analysis General population	2012–2015	22.756	VAR	R: 2–89	Global	5.5 (NM)	4.7 (NM)	2.9 (NM)	2.7 (NM)	1.6 (NM)	1.5 (NM)	NM	NM
Rosen et al. 2016 [33]	Cross-sectional General population	2010–2011	1,099	OR	X_med_:28 IQR: 21–45 R: 10–85	Peru	7.35 (NM)	10.2 (NM)	5.5 (NM)	2.24 (NM)	4.2 (NM)	1.0 (NM)	16	1) 3) 4)
Chaturvedi et al. 2015 [34]	Cross-sectional General population (NHANES cohort)	2009–2012	9,480	OR	R: 14–69	USA	6.8 (6.0–7.7)	10.5 (NM)	3.1 (NM)		6.6 (NM)	1.5 (NM)	16	1)
Hang et al. 2014 [35]	Cross-sectional General population	2009–2011	5,351	SW	X_med_:43 R: 25–65	China	0.67 (0.47–0.93)	0.67 (NM)	0.67 (NM)	0.50 (NM)	0.55 (NM)	0.46 (NM)	16	1) 3) 5)
Lang Kuhs et al. 2013 [36]	Clinical Trial Vaccinated and non-vaccinated young women	NM	5,838 (2,912 vacc.)	OR	X_med_: 26 R: 22–29	Costa Rica			Vacc. 1.6 (1.2–2.1)			Vacc. 0.7 (0.4–1.1)	51	NM
									Unvacc. 1.9 (1.4–2.4)			Unvacc. 1.3 (0.9–1.7)	16	
Pickard et al. 2012 [37]	Convenience Sample University students	2009–2010	1,000	OR	X_med_: 21 IQR: 19–23 R: 18–30	USA	2.4 (1.4–3.4)	3.2 (NM)	1.7 (NM)	1.2 (0.5–1.9)			NM	3) 5)
Gillison et al. 2012 [38]	Cross-sectional General population (NHANES cohort)	2009–2010	5,501	OR	R: 14–69	USA	6.9 (5.7–8.3)	10.1 (8.3–12.3)	3.6 (2.6–5.0)	3.7 (3.0–4.6)			16	1) 3)

Overall HPV prevalence rates (males + females) are between 0.67 and 11.89%

Limitations as stated by authors: 1) Patient history obtained from self-reports (Over - or underreporting bias); 2) Cohort partly vaccinated at enrolment; 3) Bias through initial cohort selection (patient characteristics, socio-demographic or geographic factors), low statistical power and limitations in sample size (of sub-cohorts), small number of positive oral samples and/or in detailed analysis of HPV-subtypes, 4) Possible confounders due to insufficient additional data; 5) Methodological bias (e.g. specimen collection or DNA purification method) 6) Heterogeneity of cohorts included in meta-analyses (e.g. age, region, specimen collection method etc.)

*NM* Not mentioned, *vac* vaccinated cohort, *unvacc* unvaccinated cohort

Type of sample: *OR* Oral rinse (and gargle), *SW* Oral swab, *VAR* Various (oral rinse and gargle, oral swab, brush, scraping, mucosa, saliva)

Age: X̅ - mean; X_med_ – median; R - Range; IQR – Interquartile range

^a^Oral HPV prevalence at baseline of the study

^b^Cohort size >1,000 as specified in a separate publication

^c^High risk as defined by authors of the study

Across most studies, HPV prevalence was higher in males than in females (Table 1). When not considering the studies by Hang et al. and Napolitano et al., overall HPV prevalence rates ranged between 2.3—11.89% in males and 1.6—6.2% in females in unvaccinated cohorts. The two largest cross-sectional general population cohorts were the “National Health and Nutrition Examination Survey” (NHANES) cohort from the US and the “Deep Cervical Cytology Lesions cohort” (DLCC) from China (> 9000 samples, respectively). In the various publications on the NHANES cohort oral overall HPV prevalence in the US general population was between 6.8 and 7.5% and was higher in males (10.1–11.5%) than in females (3.1–3.6%) [21, 23, 30, 31, 34, 38] (Table 1). In the Chinese DLCC cohort overall oral HPV prevalence in men (3.6%) was lower than in the US and similar in women (2.7%) [19].

Prevalence of HR-HPV varied from 0.5–4.7% in unvaccinated cohorts (Table 1). Similar to overall HPV prevalence, HR-HPV-types were also more prevalent in men than in women (0.55–7.3% vs. 0.46–1.7%). Across most studies HPV16 was the most frequently detected HR-HPV-type in unvaccinated cohorts. Only Ho et al. reported HPV52 to be the most frequently detected HR-type in adults from Hong-Kong [16].

Concerning demographic and socioeconomical data, in the NHANES cohort non-hispanic black participants were more likely (OR 1.22, 95% CI: 1.01–1.47) and Asian Americans less likely (OR: 0.33, 95% CI: 0.24–0.49) to have any oral HPV infection [31]. In the same cohort, a lower income to poverty ratio (IPR) was associated with a higher oral HPV prevalence in females (5.2% (95% CI 4.4–6.0) in the IPR < 1 population vs. 1.9% (95% CI 0.9–2.8) in the IPR > 3 population) [34].

### Oral HPV prevalence in the general population/healthy individuals – geographical differences

Interestingly, oral HPV prevalence varied in different continents and was lowest in Asia as shown in the two meta-analyses of Mena et al. (3.1%; 95% CI 0.7—6.8) and Tam et al. (2.6%; 95% CI 0.6–4.6) [24, 28]. Tam et al. found highest prevalence rates in South America (12.4%; 95% CI 5.7–19.1) followed by Europe (9.9%; 95% CI 7.2–12.5), whilst Mena et al. reported highest rates in Europe (6.5%; 95% CI 3.4–10.5) followed by North America (5.1%; 95% CI 3.6–6.8%) [24, 28]. The meta-analysis by Colpani et al., which summarized studies on oral HPV prevalence from Brazil, demonstrated an overall oral HPV prevalence of 11.8% [22] – which is consistent with the results reported by Tam et al. who found prevalence rates of 12.4% in South America [22, 28].

### Risk factors for oral HPV-DNA prevalence in the general population/healthy individuals

Established risk factors for oral HPV prevalence in the general population/healthy individuals comprise sexual behavior, concurrent genital HPV infection, smoking, and poor oral health.

Oral HPV prevalence is significantly associated with sexual behavior in both men and women. According to Gillison et al., oral HPV prevalence was eightfold higher in individuals reporting ever vs. never having sex. The study also demonstrated an increasing prevalence with lifetime or recent sexual partners (oral, vaginal or anal) with stronger association with high-risk than with low-risk HPV infections [38].

The main risk factor reported for oral HPV-DNA prevalence was the number of lifetime oral sexual partners, as shown by Pickard et al. in a cohort of 1,000 university students (OR 4.0; 95% CI 1.3–11.9) [37]. D’Souza et al. found an oral HPV prevalence rate of 0.7% in women with 0–1 lifetime oral sexual partners, 1.5% in women with ≥ 2 lifetime oral sexual partners, 1.7% in men with 0–1 and 14.9% in men with ≥ 5 oral sexual partners [74].

While some studies (e.g., Kreimer et al.) did not observe a strong association between recent sexual activity and HPV prevalence, others, including Sonawane et al. and Chaturvedi et al., found significantly higher prevalence rates in individuals reporting two or more recent oral sex partners (18.3%; 95% CI 13.0–23.5 and 9.9%; 95% CI 6.9–14.1) [30, 34, 75].

Interestingly, when it comes to oral HPV prevalence in relation to different sexual practices, a recent study by Pauli et al. with oral samples from 4,313 young adults in Brazil did not show a significant difference between those who practiced oral, anal, exclusively vaginal sex, or all of the above [75].

A further risk factor for oral HPV-DNA prevalence in immunocompetent individuals is a concurrent genital HPV infection. Oral HPV prevalence rates were 17.1% in female and 19.3% in male youths with genital HPV infection compared to 4.4% in both females and males without genital HPV infection [30, 76]. Also, having a partner with an oral or genital HPV infection is associated with higher oral HPV prevalence (7.2% overall HPV prevalence in men vs. 11.5% in men with a partner with a genital HPV infection) [77].

Oral HPV prevalence was significantly associated with smoking in both sexes in numerous studies and one meta-analysis [18, 30, 32, 78]. For example, Sonawane et al. reported a higher prevalence in smokers, particularly in heavy smokers (> 20 cigarettes/day) (23.6%; 95% CI 12.4–34.8) [30]. Contrary to prior studies, Pickard et al. found no association between smoking and oral HPV infection in a cohort of 18 - to 30-year-old students in Ohio, USA [37]. Drug use, including crack cocaine and marijuana, has also been associated with an increased oral HPV prevalence [79].

Oral health has been identified as a risk factor for oral HPV prevalence in two studies. Adults with severe periodontitis had a higher risk for oral HPV infection than those with mild or no periodontitis, as demonstrated by Ortiz et al. (OR 2.9; 95% CI 1.0–8.4) [80]. Bui et al. could also show an association between oral health and oral HPV prevalence: 10.1% (95% CI 8.2–12.4) in participants with self-reported poor to fair oral health compared to 6.5% (95% CI 5.0–8.4) in those reporting good to excellent oral health. Higher oral HPV prevalence was also shown in participants reporting that they might have gum disease, had used mouthwash to treat dental problems in the past seven days, or had higher numbers of teeth lost [81]. In contrast, two other studies found no significant association of oral HPV prevalence with periodontitis, possibly due to self-reports in contrast to clinical or radiographic diagnosis of periodontitis and differences in HPV sampling methods [82, 83].

Recently, baseline results of the MOUTH Study (Men and women Offering Understanding of Throat HPV) were published. The study includes individuals who are considered at increased risk for HPV-induced OPC due to previous sexual behavior and a history of anogenital dysplasia/cancer, either themselves or in their partners. Oral rinse samples of the 1,108 patients were tested for HR-HPV-DNA, and serum samples for antibodies against HR-HPV-oncogenes E6 and E7. HR-HPV-DNA was found in 7.3% of the participants (8.0% in men, 1.7% in women) and 22.9% had serum antibodies against HPV-oncogenes E6 or E7. At least one of these biomarkers for oncogenic HPV was found in 22.4% of the participants, a prevalence twice as high as in large cross-sectional population-based studies that only looked at oral HPV-DNA prevalence [84].

### Oral HPV prevalence in immunosuppressed patients

Human immunodeficiency virus (HIV) positivity has been established as an independent risk factor for oral HPV infection, as oral HPV prevalence rates are consistently higher in adult cohorts of PLWH than in cohorts without HIV-infection irrespective of sex, gender or sexual orientation [55, 62, 83, 85, 86]. However, comparison between studies is challenging due to heterogeneous cohort compositions—ranging from MSM (men who have sex with men) to heterosexual men and women and patients attending sexual health clinics. Thus, only a small number of studies published include data on overall oral HPV prevalence (adult men and women living with HIV) as well as on men and women separately [54, 56, 64, 66]. In Table 2 studies that report data on oral HPV prevalence in PLWH with ≥ 100 participants are summarized. All studies shown in Table 2 had a low risk of bias (Table 2).

**Table 2:** Oral HPV prevalence in people living with HIV (PLWH). Overall HPV prevalence rates in PLWH (adult males + females) are between 5 and 40%

Study	Type of study and population	Years of recruitment	No. of oral samples	Type of samples	Age (years)	Region	Overall (alpha) HPV prevalence			HR-HPV prevalence^b^			Most prevalent HR-Type^b^	Limitations^b^/risk of bias
							M + F; % (95% CI)	M; % (95% CI)	F; % (95% CI)	M + F; % (95% CI)	M; % (95% CI)	F; % (95% CI)		
Carnalla et al. 2023 [39]	(MSM-Patients)	2018	194	OR	NM	Mexico	-		-	-	14.8 (10.5–20.4)	-	16	1)
Hidalgo-Tenorio et al. 2023 [40]	Cross-sectional	NM	300	SW	X̅: 45.1 ≥ 18	Spain	13 (NM)			9.7 (NM)			16	3)
Riddell et al. 2022 [41]	Cross-sectional	2015–2017	245	OR	≥ 18	USA	23 (NM)	27 (NM)	15 (NM)	18 (NM)	23 (NM)	9 (NM)	NM	3) 4)
Perez-Quintanilla et al. 2020 [42]	Cross-sectional (Women)	2014–2015	174	SW	X_med_: 40.6	Mexico			92.5 (NM)			90.2 (NM)	51	4) 5)
Rettig et al. 2019 [43]	Cross-Sectional Cohort (HIV clinic)	2014–2015	101	SW	X_med_: 42 IQR: 37–48 R: 24–69	Cameroon	5 (2–11)			4 (NM)			68 and 82	4)
Parisi et al.^a^ 2019 [44]	Cohort (MSM; Infectious disease clinic patients)	2013	106	SW	X_med_: 44 IQR: 36–53	Italy		28.3 (NM)			9.4 (NM)		16	3) 4) 5)
Kahn et al. 2019 [45]	Clinical trial (4v vacc., MSM)	2012–2015	139	OR	X_med_: 23 R:18–26	USA		6 (NM)			2 (NM)		32/42	1) 5)
Lin et al. 2018 [46]	Cross-sectional	2013–2016	113	OR	X_med_: 26 IQR: 23–31	Taiwan					18.5 (NM)		33	4) 5)
Ablanedo-Terrazas et al. 2018 [47]	Cross-sectional	2014–2016	107	OR	X_med_: 36 IQR: 30–44	Mexico					9.3 (3.85–15.38)		NM	4) 5)
Cranston et al. 2018^c^ [48]	Clinical Trial	2012–2013	575	OR	X_med_: 47 IQR: 41–53	USA, Brazil	20						16	NM
Thorsteinsson et al. 2018 [49]	Cohort Women (SHADE cohort)	From 2011	214	SW	≥ 18	Denmark			5.6 (NM)			3.7 (NM)	52	6)
Vergori et al. 2018 [50]	Cross-sectional	2015–2016	305	SW	≥ 18	Italy		20.9 (NM)					66	1) 5)
Steinau et al. 2017[51]	Cohort	2012–2014	110	OR	18–26	USA		21.8 (NM)					16^e^	1) 5)
King et al. 2016[52]	Systematic review Meta-analysis MSM	1997–2015	2886	VAR	NM	World		28.9 (19.1–38.7)			16.5 (8.2–24.8)			NM
Moscicki et al. 2016[53]	Cross-sectional Paediatric (PHACS cohort)	NM	209	OR	Children	USA	2 (0.5–4.8)	3 (NM)	1 (NM)				NM	3)
Shiboski et al.^a^ 2016 [54]	Observational	2010–2012	388	OR	≥ 18	USA	18 (14–22)	20 (16–25)	11 (5–20)	5 (3–7)	4 (2–7)	5 (1–13)	16	6)
Vacharotayangul et al. 2015 [55]	MSM + Heterosexual Females		187	OR		Thailand	17.2	25	9.4					
Kahn et al. 2015 [56]	Cohort Convenience sample (clinics)	2011–2012	272	OR	12–24	USA	19.5 (9.7–30.9)	19.7 (7.6–15.4)	18.6 (7.7–16.8)	11 (2.8–18.7)	11.7	8.5	M: 16 and 59 F: 52	3) 4) 6)
Gaester et al. 2014 [57]	Cohort	2011–2013	283	OR	22–72	Brazil		3.5 (NM)					66	NM
Darwich et al.^a^ 2014 [58]	Cohort	NM	650	OR/SW	NM	Spain		16 (NM)					16 (NM)	5)
Lima et al. 2014[59]	Cohort Convenience sample (clinics) Partly w/lesions	NM	100	SC	32–52	Brazil			11 (NM)			7 (NM)		NM
Beachler et al. 2013 [60]	Cross-sectional Convenience sample	2006	404	OR	NM	USA	28 (NM)			13 (NM)			NM	5) 6)
Videla et al. 2013 [61]	Cohort	2005–2009	764	OR/SW	≥ 18	Spain		MSM 16 (13–19)					16	5) 6)
								Het. 19 (13–25)						
Mooij et al. H2M Cohort 2013 [62]	Cohort	2010–2011	314^c^	OR	≥ 18	Nether-lands		56.7 (51.2–62.2)			24.8 (20.0–29.5)		16	4) 5)
Marchetti et al. 2013 [63]	Cross-sectional	2009–2011	277	CU	≥ 18	Italy	20 (NM)			9 (NM)			16	3)
Beachler et al. 2012 [64]	Cross-sectional	2009–2010	379^c^	OR	NM (adults)	USA	40 (NM)	45 (NM)	35 (NM)	21 (NM)	23 (NM)	18 (NM)	16	1) 3) 5) 6)
Read et al. 2012 [65]	Cross-sectional (MSM)	2010	249^c^	OR/TA/SW	≥ 35	Australia		19 (15–25)			8 (5–12)		16	1) 5)
Del Mistro et al. 2012 [66]	Cross-sectional	2007–2010	100	SA	NM	Italy	37 (NM)	MSM 42.1 (NM)	31.6 (NM)	13 (NM)	MSM 18.4 (NM)	2.6 (NM)	16	NM
								Non-MSM 37.5 (NM)			Non-MSM 20.8 (NM)			

Age: X̅—mean; Xmed – median; R—Range; IQR – Interquartile range

Limitations as stated by authors: 1) Patient history obtained from self-reports (over - or underreporting bias); 2) Cohort partly vaccinated at enrolment; 3) Bias through initial cohort selection (patient characteristics, socio-demographic or geographic factors), low statistical power and/or limitations in sample size (of sub-cohorts), small number of positive oral samples and/or in detailed analysis of HPV-subtypes; 4) Possible confounders due to insufficient additional data 5) Methodological bias (e.g. specimen collection or DNA purification method) 6) Heterogeneity of cohorts included in meta-analyses (e.g. age, region, specimen collection method etc.)

Type of sample: OR Oral rinse (and gargle), SW Oral swab, SC Oral scraping, CU Oral Curettage, TA Tampon, SA Saliva, VAR Various (oral rinse and gargle, oral swab, brush, scraping, mucosa, saliva)

*NM *Not mentioned

^a^Only baseline data included from studies with follow-up or incidence data

^b^As defined by authors of the study

^c^Size of HIV sub-cohort

^d^prevalence only measured for 9v vaccine types

^e^not specific for HIV cohort

**Fig. 3:**
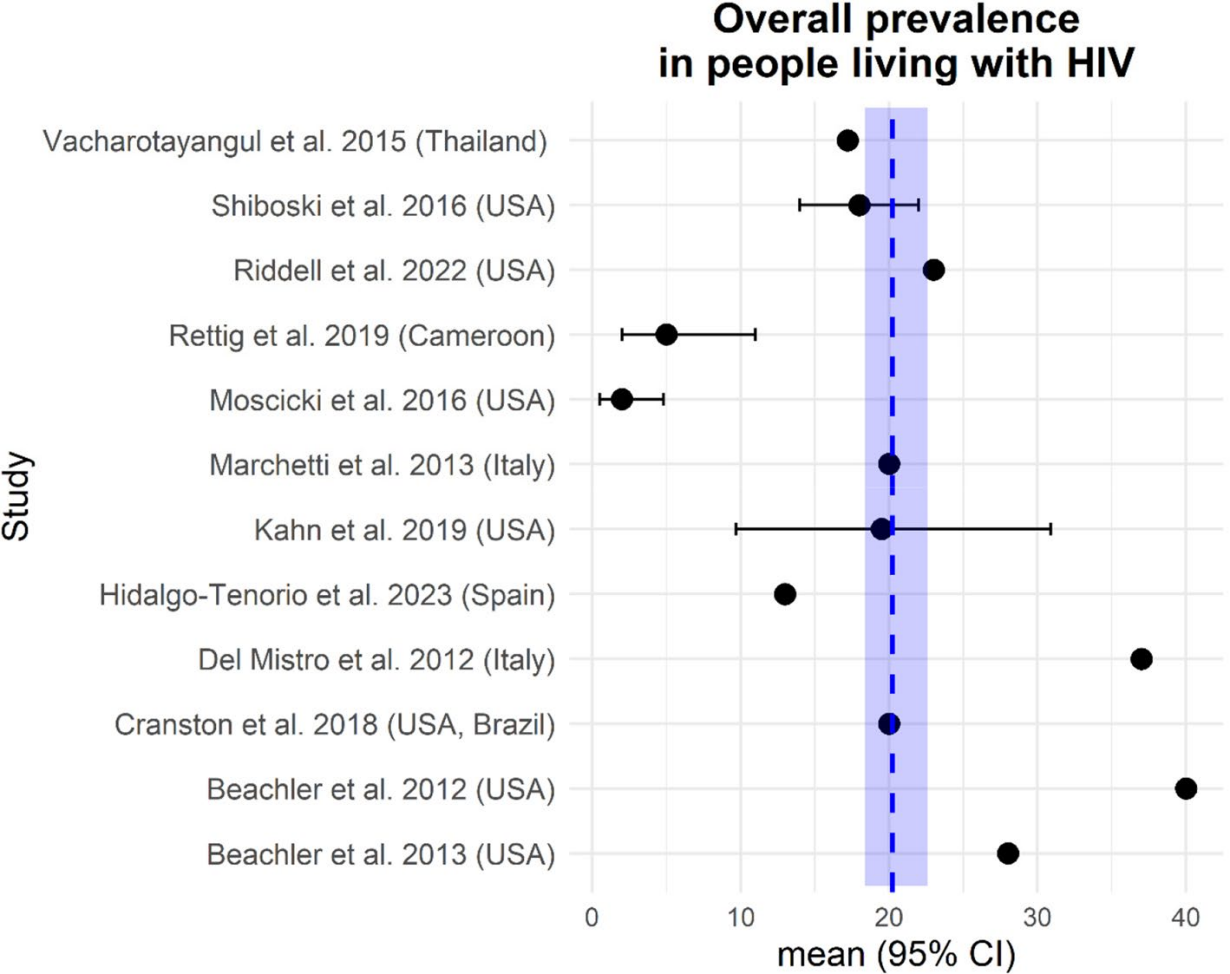
Forrest-plot summarizing the overall oral prevalence from studies (2012–2023) with PLWH reporting data on males and females. The blue dotted line represents the mean prevalence over all studies. The shaded blue area marks the upper - and lower confidence interval. The study by Moscicki et al. (2016) covers children that were perinatally infected with HIV

Studies reporting overall oral HPV prevalence in mixed-gender adult PLWH cohorts show a wide range of 5–40% (Table 2, Fig. 3), reflecting substantial methodological differences and/or distinct populations. Figure 3 shows all studies listed in Table 2 that report data on overall HPV prevalence in female and male PLWH [45]. [insert Fig. 3] In general, oral HPV prevalence is higher in adult men with HIV-infection than in women living with HIV (Table 2). However, reported rates vary dramatically—from rates close to the general population (e.g. 6% or 3.5% in males) to rates much higher than in the general population (e.g. 56.7% or 45% in males) [45, 57, 62, 64]. Unusually high prevalence rates of 92.5% for oral HPV and of 90.2% for oral HR-HPV were reported in Mexican females with HIV-infection by Perez-Quintanilla et al. [42]. These results are in strong contrast to studies on oral HPV prevalence in males living with HIV from Mexico [39, 42] (Table 2) and therefore should be interpreted with caution. Across all other studies prevalence data concerning female adults with HIV-infection ranged between 5.6–35% (Table 2) [49, 64]. Perinatally HIV-infected children have a much lower oral HPV prevalence (2% (95% CI 0.5–4.8)) than adult PLWH [53].

Most data on oral HPV prevalence in PLWH have been collected in cohorts of MSM [39, 44, 45, 55, 85]. When looking at data gathered exclusively from MSM-cohorts, oral HPV prevalence rates seem higher among MSM than in other populations (12% in HIV-negative MSM in Cotte et al.; 15% in HIV-negative MSM and 21.2% in MSM with HIV in Giuliani et al.; 17.3% in HIV-negative MSM and 27.8% in MSM with HIV in Rollo et al.; 27.6% in HIV-negative MSM and 56.7% in MSM with HIV in Mooij et al.) [62, 87–89]. However, this could not be confirmed in a meta-analysis of King et al., who found no difference (pooled OR 1.07, 95% CI 0.65–1.74) regarding the effect of identifying as MSM on oral HPV prevalence compared to heterosexual men [52].

A recent Spanish study of 300 MSM and women with HIV identified HPV16 as the most common type and found coinfection with *Treponema pallidum* and a history of anal high-grade dysplasia or cancer as risk factors for oral HPV. Longer duration of antiretroviral therapy (ART) was considered a protective factor [40].

There is very few data on oral HPV prevalence in solid organ transplant recipients (SOTR). In a study from 2001 investigating oral HPV prevalence in PLWH and SOTR with small patient numbers (57 and 40 participants), HPV-DNA was detected in the cytobrushings of 7.0% of PLWH and 20% of SOTR [90]. The largest study to date, comparing 88 renal transplant recipients (RTR) with 88 healthy controls found HPV-DNA using oral scrapings in 18% of RTR and in 1% of controls [91]. In concordance with this, a small Polish study using oral brush sampling also found a high oral HPV prevalence of 28% in 32 SOTR, albeit no control group was analyzed [92]. However, a recent study from Denmark showed no overall difference in oral HPV prevalence between 250 RTR and 250 controls (12.1% vs. 10.4%), though female RTR trended to have a higher oral HPV prevalence than controls (OR 1.73, 95% CI 0.63–4.77) [93].

### (Type-specific) persistent oral HPV infection

Similar to anogenital HPV infection and anogenital cancer, it is assumed that persistent oral infections with oncogenic HPV-types are the driving force behind HPV-associated OPC, with HPV16 infection being the dominant type [94, 95]. However, the exact patterns of oral HPV persistence remain unclear and analyses regarding the natural history of oral HPV infection show differences across anatomical sites [96]. Long-term (> 4–6 years) studies on oral HPV persistence either in the general population or in PLWH are still scarce and methodological differences among the existing studies cause difficulties when trying to review the data. Main challenge are, that “persistence” is defined differently across different studies and that standardized measurement frequencies and fixed time intervals regarding clearance of oral HPV-DNA do not exist. The most recent meta-analysis of Tam et al., for example, did not evaluate persistence because of different reporting methods for persistence and clearance of oral HPV infections across different studies [28]. Below we summarize data on oral HPV-DNA persistence, bearing in mind that the studies are only comparable to a limited extent due to different methodological approaches.

Reported (type-specific) persistence rates in healthy individuals vary widely. U.S.-based studies show persistence rates ranging from 0% (Cook et al.) to 39% (Pickard et al.) [37, 97], while in an all-female, partly vaccinated Costa Rican cohort, 17.6% of alpha-HPV infections persisted over two years, including 15.4% for carcinogenic HPV-types [98]. In Finland, a 6-year follow-up study showed that 74 of 324 (22.8%) women had persistent oral HPV infection determined as at least two consecutive samples positive with the same HPV-type [99].

The HPV infection in men (HIM) study demonstrated 24-months persistence rates for different oral HR-HPV-types between 7–32%. HPV39 had the highest 24-months persistence rate (31.8%), followed by HPV31 (30%) and HPV56 (23.5%). Persistence of HPV16 was found in 18% of the men; the median time for clearance of HPV16 was 6.4 months. Older age was identified as a key risk factor for prolonged persistence [26]. Another publication on the HIM cohort demonstrated that oral HPV16 infections in men present at baseline were significantly more likely to persist than incident infections. At 24 months, 80% of baseline infections persisted compared to 10% of incident infections; at 48 months the respective persistence rates were 40% for infections present at baseline vs. 0% for incident infections [100].

For a better understanding of the longitudinal dynamics and associated risk factors of oral HPV infection, an incidence and clearance study with frequent testing was recently published by Brouwer et al.. Concerning HPV clearance, only 16% of detected genotypes persisted to the next study visit, which resulted in a mean time to clearance of a genotype of 46 days [101]. Among PLWH higher oral HPV incidence and persistence rates are frequently found [102]. Similarly to the non-HIV population, less incident than prevalent infections persisted for at least two years (7% vs. 35%) [102]. In a recent U.S. study by Riddell et al., PLWH had a higher prevalence of any tested HPV genotype at baseline and showed longer time to clearance (mean 473 days (95% CI: 336, 664)) compared to HIV-negative controls (97 days (95% CI: 62, 151)). Immune dysfunction, non-adherence to ART and a history of AIDS were associated with longer time to clearance, although not reaching significance [41]. A study with 173 Australian MSM living with HIV found a notably high 3-year persistence rates of 47% (95% CI 28–66) for any HPV detected at baseline and 40% (95% CI 16–68) for HR-HPV-types. Persistence was associated with duration of HIV-infection (OR 1.13 per additional year) and tonsillectomy (OR 8.17; 95% CI 1.30–51.40). Interestingly, sexual behaviour variables were not statistically associated with persistence [103].

### HPV localization in the oral cavity and prevalence in non-malignant tonsils

In anogenital cancer, persistent HR-HPV infection can cause precursor lesions (high-grade cervical, vulvar, vaginal, anal or penile dysplasia), which can progress to invasive carcinomas [46, 104, 105]. In HPV-associated OPC, such precursor lesions have not yet been identified [106, 107]. Notable differences in the natural history of the cervical vs. the oropharyngeal HPV infection, such as the frequency of HPV-DNA integration, have been established [107]. The tonsils and the base of the tongue are the predominant locations of HPV-associated OPC, possibly because the mucosal junction cells and the discontinuous epithelium make the basal epithelium more easily susceptible to HPV infection [107]. Also, the deep tonsillar crypts may serve as a reservoir for HPV and the surrounding lymphoid tissue could be instrumental in creating an immune-privileged site as well as allowing tumors to evade immune surveillance through programmed cell death protein 1 (PD-1)/programmed cell death-ligand-1 (PD-L1) receptor [108–110]. Rieth et al. have shown the co-localization of the HPV capsid protein L1 to the bacterial biofilm in tonsillar crypts by immunohistochemical analysis of non-malignant HPV-positive tissue. Also, extranuclear presence of the viral capsid protein L1 could be demonstrated, possibly indicating viral shedding [111]. Nevertheless, HPV prevalence in non-malignant tonsillar tissue is low. A systematic review of pediatric tonsillectomy samples found HPV-DNA in 0–21% of cases [112], with HPV prevalence rates being below 10% in 9 of the 11 studies included. Several large studies from the UK, USA, Brazil, China, and Canada reported a HPV prevalence rate of 0% [112–114]. In non-malignant adult tonsil samples HPV prevalence is similarly low and ranges between 1–6.8%. However, differences in study design such as methods used for HPV-typing do not allow a direct comparison of the studies examined [111, 115, 116]. In one small study, HR-HPV-DNA-load found in non-malignant tonsils was very low compared to HPV-associated OPC [117]. The prevalence of HPV in tonsils seems to be much lower than in oral rinses or swabs. In a recent Swedish study comparing tonsillectomy biopsies to mouthwash samples, collected at the time of surgery, none of the tonsils were HPV-positive, despite an HPV prevalence of 10.3% in the corresponding mouthwash samples [118]. In addition, a history of tonsillectomy was not significantly associated with the presence of HPV in oral rinse samples according to a recent publication of Wu et al. based on the NHANES cohort [119].

### The effect of HPV vaccination on oral HPV infection

Although HPV vaccines are considered to be highly effective against the HPV strains mostly found in the oropharynx, no studies have been published that directly show that prophylactic HPV vaccination can prevent HPV-associated OPC [120].

A preclinical investigation demonstrated the prevention of oral HPV16 pseudovirus infection in mice by prophylactic immunization with HPV vaccines licensed for humans [121]. A clinical study with 27 to 45-year-old US males demonstrated that most HPV-vaccine recipients developed HPV16 and 18 antibodies detectable in oral gargles. Antibodies against both HPV-types were still detectable 18 and 30 months after vaccination, albeit in a lower number of participants (29.6% at 30 months for HPV16) than 7 months post-vaccination (93.2% for HPV16) [122, 123].

In the previous decade, increasing evidence was generated showing that HPV vaccination can prevent oral HPV infection [69, 124, 125]. A study conducted among 1,784 high school students in Colombia showed a much lower oral HPV prevalence in vaccinated girls (0.7%) than in unvaccinated girls (3.2%) and unvaccinated boys (2.3%). The difference was almost ten-fold compared to sexually active boys [27]. A randomized blinded HPV vaccine trial among 7,466 women in Costa Rica showed an estimated 93.3% vaccine efficacy against oral HPV16/18 infection and a 91.6% type-specific efficacy against HPV16 [126]. Results of the US NHANES study, further support that oral HPV prevalence of vaccine genotypes (HPV6,11,16,18) is lower in vaccinated than in unvaccinated cohorts, although the number of vaccinated males is small throughout the studies [124, 127, 128]. In Table 3 all studies from Table 1 and 2 are summarized that report type-specific oral HPV prevalence of the nonavalent vaccine genotypes. The prevalence of HPV vaccine genotypes varies between 0 and 20%, depending on vaccination and HIV-status (Table 3). In the few studies that compared vaccinated and unvaccinated cohorts oral HPV prevalence of vaccine genotypes is lower in vaccine recipients than in unvaccinated individuals supporting the findings of the US NHANES results mentioned above [126, 128, 129]. In a large randomized vaccine trial conducted between 2007 and 2014 in Finland, a high efficacy of the AS04-HPV16/18 vaccine (Cervarix®) against oropharyngeal infections with HPV16 and 18 and a moderate efficacy against HPV-types 31, 33 and 45 was shown. No efficacy was found against other HR-HPV-types [129].

**Table 3:** Type-specific oral HPV prevalence of the nonavalent vaccine types in % (studies from Table 1 and 2)

Study	Groups analysed	HPV6	HPV11	HPV16	HPV18	HPV31	HPV33	HPV45	HPV52	HPV58	Total HR prevalence	Total prevalence	Prevalence of HPV vaccine types	% of HR covered by vaccine
General population														
Giuliano 2023 [18]	Vacc	0	0	1.1	0	NM	NM	NM	NM	NM	NM	NM	1.1 (4v) 1.1(9v)	
	Unvacc	0.2	0	0.7	0.3	NM	NM	NM	NM	NM	NM	NM	1.2 (4v) 1.6 (9v)	
Schlecht 2019^a^ [25]	Vacc	0.2 ^b^	0.2 ^b^	0.1	0.1	0	0.2	0	0.1	0.3	1.7	6.2	0.4 (4v) 1.0 (9v)	
	Unvacc	1.05 ^b^	1.05 ^b^	1.05	0	0.5	0	0	0	0			2.1 (4v) 2.6 (9v)	
Mena 2019^a^ [24]		0.2	0.1	1.0	0.2	NM	NM	NM	NM	NM	2.6	4.9	NM	
Bettampadi 2019 HIM cohort [26]	Brazil	1.1	0.2	1.7	0.7	0.2	0.1	0.2	0.8	0.3	5.3	8.7	3.5 (4v) 4.5 (9v)	64 (9v)^a^
	Mexico	1.3	0.6	1.8	0.5	0.4	0.0	0.5	0.5	0.3	7.3	10.0	4.0 (4v) 5.5 (9v)	38 (9v)^a^
	USA	0.5	0	1.6	0.2	0.2	0.5	0.1	0.8	0.1	5.4	7.6	2.2 (4v) 3.7 (9v)	41 (9v)^a^
Castillo 2019 [27]	Total	0.06	0	1.18	0	0	0.06	0	0	0	NM	1.51	NM	
	Vacc. girls	0	0	0.53	0	0	0.11	0	0	0				
	Unvacc. girls	0	0	2.11	0	0	0	0	0	0				
	Unvacc. boys	0.13	0	1.18	0	0	0.06	0	0	0				
Sonawane 2018 NHANES cohort^a^ [30]	Men	0.7	0.1	1.8	0.5	0.3	0.4	0.2	0.4	0.3	7.3	11.5	Vacc. 0.18 (4v) Unvacc. 1.47 (4v)	
	Women	0.1	0	0.3	0	0	0	0.1	0.1	0	1.4	3.2		
Rosen 2016^a^ [33]		1.5	0	1.2	0.1	0.2	0	0.1	0.2	0.4	2.24	7.35	NM	
Hang 2015 [35]		0.02	0.06	0.43	0	0	0	0.07	0	0.02	0.50	0.67	NM	
Lang Kuhs 2013 [36]	Total	0.1	0.02	0.2	0.1	0.1	0	0	0.2	0.02	1.0	1.7	NM	
	Vacc	0.1	0.03	0.03	0	0.1	0	0	0.1	0	0.7	1.6		
	Unvacc	0.1	0	0.4	0.1	0.2	0	0	0.2	0.03	1.3	1.9		
Gillison 2012^a^ [38]		0.38	0.02	1.0	0.22	0.13	0.14	0.27	0.22	0.11	3.7	6.9	NM	
HIV-population														
Parisi 2019 [44]		NM	NM	2.83	0.94	0.94	0	0	0	0.94	9.4	28.3	NM	
Kahn 2019 [45]		0	0	0	0	0	0	0	0	0	2	6	0 (4v) 0 (9v)	0 (9v)^a^
Cranston 2018^d^ [48]		2	2	5	2	3	4	1	0	3	NM	NM	10 (4v) 20 (9v)	
Thorsteinsson 2018 [49]		0.5	0.5	0.5	0	0.5	0	0	1.4	0.5	3.7	5.6	NM	12.5 (4v) 50 (9v)
Vergori 2018^a^ [50]		0.3	1.6	1.0	0.3	0	1.6	0.3	0	0.7	NM	20.9		26.6 (9v)°
Shiboski 2016^a^ [54]		NM	1	14	NM	2	NM	NM	NM	NM	5	18	NM	
Darwich 2014^a^ [58]	MSM	2.5	0.2	3.8	0.2	0.2	2.6	1.8	0	NM	NM	16	NM	
	Het	2.0	0.4	6.8	0	0.4	2.0	0.4	0	NM				
Mooij 2013^a^ [62]	HIV + MSM	1.9	1.8	5.4	2.2	2.8	4.8	1.6	2.2	1.4	24.8	56.7	10.2	
	HIV—MSM	0.9	0.4	2.0	0.6	0.2	0.8	0.2	0.8	0	8.8	27.6	3.5	

^a^as deduced from figures or data if not mentioned in the text of the original publication

^b^common Data for HPV6/HPV11

conly 9v vaccine types measured in study; °—low risk and high risk; NM – not mentioned; 4v – quadrivalent vaccine; 9v – nonavalent vaccine

In a study from 2022 with participants of the NHANES cohort, an HPV prevalence of 7.21% (95% CI 6.29–8.26) was found in the unvaccinated female and male cohorts, compared to 4.22% (95% CI 3.23–5.52) in vaccinated individuals. Males had a higher oral HPV prevalence than females, irrespective of vaccination status [21].

### Sampling methods for the detection of oral HPV infections

Although many studies have investigated oral HPV prevalence, few studies have assessed the optimal sampling method for accurate HPV detection. The existing studies are summarized in Table 4. Most studies used oral rinses/gargles or oral swabs (Tables 1 and 2), but comparative data on the sensitivity of the sampling methods are limited. Evidence suggests that oral rinse or gargle samples are generally the most sensitive and practical option. A study conducted on 100 cancer-free MSM showed a higher HPV detection rate in oral rinse samples compared to oropharyngeal or oral cytobrushings [69]. Similar results were shown in a healthy adult population: in a study comparing mouthwash gargles, tonsil brushings and snap frozen tonsillar biopsies, the highest HPV prevalence was found in the mouthwash gargles [72]. Both studies demonstrated poor agreement in HPV-positivity between the different sampling methods [69, 72]. A Japanese study found no significant benefit in adding tonsillar brushing to oral gargling, suggesting that gargles alone may be sufficient for screening purposes [125].

**Table 4:** Oral HPV prevalence in studies analyzing different sampling methods

Study	Type of study and population	Years of recruitment	No. of samples included	Age (years)	Region	Type of samples compared	Overall (alpha) HPV prevalence			Results					Limitations
							M + F; % (95% CI)	M; % (95% CI)	F; % (95% CI)	Pos. agr.; %		Ovr. agr.;%		Cohen K (95% CI)	
Castaneda-Avila et al. 2022 [68]	Cross-sectional	2014–2016	346	40–65	Puerto Rico	OR	4.3							0.61 (0.45–0.78)	2)
						OS	5.8								
Dona et al. 2019 [69]	Case series from longitudinal study (MSM-Patients, partly HIV-pos. OHMAR cohort)	2014–2018	162	≥ 18	Italy	OR		58.9 (NM) HR: 30.2 HPV16: 9.9		16.8 OR vs. OPB 14.3 OR vs. OB		51.2 OR vs. OPB 52.0 OR vs. OB		0.14 (0.07–0.21) OR vs. OPB 0.13 (0.04–0.21) OR vs. OB	1) 2)
						OPB		9.9 (5.6–16.0) HR: 5.5 HPV16: 3.1							
						OB		8.0 (3.5–15.8)							
Chikandiwa et al. 2018 [70]	Cohort (HIV-pos. men)	NM	181	23–62	South Africa	OR		1.8 (0.4–5.1)		6		82		0.09 (NM)	1) 3)
						OS		0.6							
de Souza et al. 2018 [71]	Cohort (GP clinic patients)	NM	96	20–70	Australia	OR, single GP +		10.4		NM		88 OR vs. SA GP + 84 OR vs. SA Nested		0.11 (−0.15–0.37) 0.36 (0.10–0.61)	2)
						SA, single GP +		3.1							
						OR, Nested PCR		11.5							
						SA; Nested PCR		16.7							
Combes et al. 2017 [72]	Cross-sectional (Benign tonsillectomy patients SPLIT cohort)	2012–2016	692 TB (incl. children)	1–70	France	TB	3.6 (NM)	4.5 HPV16: 2.2	2.9 (NM)	9.5		85.8		NM	2) 3) 4)
			268 OR			OR	13.1 (NM)	18.2 HPV16: 4.1	8.8 (NM)						
Steinau et al. 2012 [73]	Cohort (HIV patients)	2009	100		USA	OR	39					90.2% Type specific concordance			1) 3)
						MB	13								
						TB	15								

Oral rinse and gargle is the most widely used sampling method

Limitations as stated by authors:

1) Bias through initial cohort selection (patients characteristics, socio-demographic or geographic factors)

2) Methodological bias (e.g. Sample DNA not quantified, viral load not measured, positive samples not typeable, technical difficulties in sample collection)

3) Low statistical power, limited sample size (of certain sub-cohorts), small number of positive oral HPV infections and limitations in detailed analysis of HPV-subtypes

4) Possible confounders due to insufficient additional data

*NM* Not mentioned. Type of sample: OR Oral rinse (and gargle), OPB Oropharyngeal brushing, OB Oral brushing, SW Oral swab, SA Saliva, TB Tonsil brushing, MB Mucosal brushing

According to Read et al. oral rinse with saline has the highest sensitivity (97%) for the detection of oral HPV, followed by tampon-absorbed oral rinses (69%) and oral swabs (32%) [65]. Similarly, in a study on PLWH, all individuals with HPV detected in mucosal, tonsil or lesion brushings were also positive in their oral rinse sample. This led the authors to conclude that oral rinse provides the best representation for HPV in the oral cavity [73]. However, contrasting results from Castaneda-Avila et al. showed lower detection rates with oral rinse compared to cytobrush, possibly due to cohort-specific factors or differences in sampling techniques [68].

It is still unclear, however, which sampling method is most representative for the detection of clinically relevant prevalent and persistent oral HPV infections. A higher detection rate is not necessarily indicative of a clinically relevant oropharyngeal HPV infection. Although oral rinse and gargle is currently the most frequently used method for oral sampling, it does not provide a targeted sample of the oropharynx. Nonetheless, oral rinse and gargle seems to be the most suitable method to determine oral HPV-DNA prevalence.

## Discussion

HPV-associated OPC is an increasing sub-entity of head and neck squamous cell carcinoma, with HPV16 being the most prevalent type found in the tumors [130, 131]. It has been shown that oral HPV infection is involved in the pathogenesis of theses cancers [132, 133]. However, only limited current data on worldwide oral HPV prevalence exist. In the period we examined (2012–2024), five systematic reviews were published that summarize the current data on HPV prevalence – four in the general population and one in MSM. Three of the above-mentioned reviews summarized studies, of which some date back until 1995: Tam et al. (1995–2017) [28], Mena et al. (1995–2015) [24] and King et al. (1997–2015) [52]. There is a risk that the inclusion of studies published three decades ago will not accurately represent the current average oral HPV prevalence. The present review also comprises more recent studies with participant recruitment up to 2023. It contributes updated prevalence data across different populations, confirming lower oral HPV prevalence rates in Asia compared to North America, and highlighting significant variations by geographical region, age, sex, and HIV status [22, 24, 28, 52].

Little is known about the mechanism by which oral HPV infection induces OPC. In the literature, this is mainly discussed in relation to cervical cancer, where persistent HR-HPV infection is the main cause of cancer development [132–134]. In our review we report type-specific persistence rates in healthy individuals ranging from 0 to 39%, with the largest study showing 24-months persistence rates between 7 and 32% in unvaccinated males, depending on the HR-HPV-type found. For HPV16, the most frequent type found in OPC, the 24-months persistence rate was 18% [26, 37, 135]. In PLWH, often higher persistence rates are reported than in other at-risk cohorts [102, 103]. The wide variability in persistence rates reflects methodological differences across studies, but also points to the need for standardized protocols and more long-term, multicenter research, particularly among immunocompromised and understudied populations. Most persistence studies to date originate from the Americas, with a notable lack of data from Asia, Africa and other underrepresented regions. Standardized, global, longitudinal studies are needed to clarify natural history, particularly for high-risk HPV-types such as HPV16 [28].

We furthermore identified a lack of standardization of sampling methods for HPV detection in the oral cavity. Currently, the most frequently used sampling method is oral rinse and gargle [68, 73, 130]. By oral rinse/gargle sampling it cannot be determined where the detected HPV-DNA was located (oropharynx vs. oral cavity). Studies analyzing different sampling methods are scarce (see Table 4). Up to date the majority of studies conclude that oral rinse/gargle provides the best representation of HPV in the oral cavity and oropharynx [68, 73, 130]. In future studies, a standardized methodological approach for sample collection should be aimed at.

Concerning the efficacy of HPV vaccination for the prevention of HPV-associated OPC, no studies have yet been published that demonstrate a decline in OPC incidence. However, a report from the American Society of Clinical Oncology (ASCO) 2024 annual meeting summarized a not yet published large US database study that showed that men who received the HPV vaccine had a 56% lower risk for head and neck cancers than unvaccinated men (OR, 0.44; *P* < 0.001) [136]. Prophylactic vaccination, particularly in HPV-naïve adolescents, appears to significantly reduce oral HPV infection, supporting its role in cancer prevention strategies [27, 127]. In 2020, mounting evidence for vaccine efficacy concerning prevention of oral HPV infection resulted in the FDA approval (accelerated approval licensure pathway) of the nonavalent HPV vaccine Gardasil9® in the USA for the prevention of OPC [137]. However, it should be noted that the approval was based on immunological data, not on data about OPC prevention. As mentioned above, no studies have yet been published that demonstrate a decline in OPC incidence following the introduction of HPV vaccination. With over 140 countries now including HPV vaccination in their national immunization programs, a global decline in HPV-related cancers is anticipated [136].

This review is not without limitations. Study heterogeneity and language restrictions may have led to selection bias. The inclusion thresholds for study size (> 1000 participants in the general population/healthy individuals; > 100 in PLWH) may have excluded smaller but relevant studies, particularly in high-risk groups (such as MSM without HIV-infection). For the risk of bias assessment, the STROBE checklist was used, which is not established for measuring risk of bias in meta-analyses. When further addressing concerns about study quality, some studies were excluded due to poor methodology and reporting, leading to a possible reporting bias. Nonetheless, the excluded studies lacked sufficient information about sampling or outcome, thus hampering the interpretation of their results. Standardization of the definitions of HPV clearance and persistence is needed for conducting future systematic reviews and meta-analyses on this topic.

This systematic review has several strengths. Not only were recent large studies relating to the general population included, but also studies conducted in PLWH and SOTR. Additionally, attention was brought to the effect of prophylactic vaccination on oral HPV prevalence. Furthermore, possible differences in study outcomes when using different sampling methods were reported. This has led to a broad and comprehensive review of the natural history of HPV infection of the oral cavity and the tonsils.

In summary, oral HPV is prevalent in populations worldwide, particularly in males of the general population, in PLWH, and in SOTR. HPV vaccination offers a powerful tool for primary prevention of oral HPV infection. Improving vaccination coverage and expanding research into long-term infection dynamics and standardization of sampling and detection methods for oral HPV infection will be crucial for the reduction of the global burden of HPV-associated OPC.

## Conclusions

In this review we could present differences in oral HPV prevalence across various population groups by gender, age, HIV status, and geographic region. Oral HPV-DNA can be found in 1–12% (mean 5%) of the general population, more frequently in men than in women with most infections being transient. Risk factors for oral HPV infection comprise the number of lifetime (oral) sex partners, male sex, smoking, oral health, older age and concurrent genital HPV infection. Oral HPV prevalence is higher in adult PLWH than in the general population. Although HIV is an independent risk factor for oral HPV infection, pediatric patients living with HIV have a lower oral HPV prevalence than the general population. Prophylactic HPV vaccination is associated with a significant reduction in vaccine-type oral HPV prevalence. Therefore, high vaccination rates in children and adolescents are crucial to counteract the rise in HPV-associated OPC in the future. Identifying differences in oral HPV prevalence systematically might support the development of targeted prevention strategies. However, there is still a need to include more underrepresented groups (like MSM without HIV and SOTR) in future studies on oral HPV prevalence. Finally, we identified a lack of standardization of sampling methods for HPV detection in the oral cavity. In future studies on oral HPV prevalence, a standardized methodological approach for oral sample collection should be applied.

## Data Availability

The datasets used to create Tables 1 and 2 and Figs. 2 and 3 are available from the corresponding author on reasonable request.

## Abbreviations

ART: Antiretroviral therapy
CO: Oral Curettage
HPV: Human papilloma virus
HR: High-risk
MB: Mucosal brushing
MSM: Men who have sex with men
MSMLWH: Men who have sex with men living with HIV
n.a.: Not applicable
NHANES: National Health and Nutrition Examination Survey
NM: Not mentioned
OB: Oral brushing
OPB: Oropharyngeal brushing
OPC: Oropharyngeal cancer
OR: Oral rinse (and gargle)
OS: Overall survival
PLWH: People living with HIV
PRISMA: Preferred Reporting Items for Systematic Review and Meta-Analysis
R(C)T: Radio(chemo)therapy
RTR: Renal transplant recipients
SA: Saliva
SC: Oral scraping
SOTR: Solid organ transplant recipients
SW: Oral swab
TA: Tampon
TB: Tonsil brushing
